# Risk Factors for Increased Online Gambling during COVID-19 Lockdowns in New Zealand: A Longitudinal Study

**DOI:** 10.3390/ijerph182412946

**Published:** 2021-12-08

**Authors:** Maria E. Bellringer, Nick Garrett

**Affiliations:** 1 Gambling and Addictions Research Centre, Faculty of Health and Environmental Sciences, Auckland University of Technology, Auckland 1142, New Zealand; 2 Department of Biostatistics and Epidemiology, Faculty of Health and Environmental Sciences, Auckland University of Technology, Auckland 1142, New Zealand; nick.garrett@aut.ac.nz

**Keywords:** COVID-19, online gambling, New Zealand, longitudinal, pandemic, lockdown

## Abstract

Recent research investigating changes in gambling behaviors during periods of COVID-19 social restrictions, such as enforced lockdowns, are somewhat limited by methodology, being generally cross-sectional in nature and with participant samples recruited via online panels. The present study overcame these limitations via a secondary analysis of data collected in 2012 and 2015 from a New Zealand (NZ) longitudinal gambling study, with questions related to gambling behaviors due to COVID-19 lockdown periods included in an additional data collection, of participants who had previously scored as a risky gambler, during 2020/21. Almost one-quarter of online gamblers increased their gambling during lockdown with this most likely to be on overseas gambling sites, instant scratch card gambling and Lotto. The only sociodemographic risk factor for increased online gambling was higher education. Behavioral risk factors included being a current low risk/moderate risk/problem gambler, a previously hazardous alcohol drinker or past participation in free-to-play gambling-type games. These past behaviors could act as trigger points for health services or family and friends to monitor a person’s gambling behaviors during lockdown, or future stressful periods when usual terrestrial gambling opportunities are curtailed or unavailable, and to support safer gambling practices.

## 1. Introduction

New Zealand (NZ) is a small geographically isolated country located in the Pacific Ocean, with a population of approximately 5 million people [1]. The first COVID-19 case was reported in NZ on 28 February 2020 [2]. Less than four weeks later, the country moved into self-isolation entering Alert Level 3 on 23 March and then Level 4 on 25 March. In effect, both Level 3 and Level 4 were states of national lockdown with people instructed to stay home in a household ‘bubble’ and not to integrate with other household bubbles. Level 3 restrictions allowed businesses to operate, but only in a contactless manner with social distancing and hygiene guidelines. In Level 4, people could only leave home to access essential services or for exercise in the local area, maintaining social distancing from others. Only essential services could operate (i.e., health and emergency services, pharmacies, supermarkets and petrol stations) with all other client-facing services closed. Online services could operate under strict social distancing and hygiene rules [3]. This state of lockdown continued until 13 May when the Alert was reduced to Level 2 and services could again be accessed with social distancing, then finally a move to Level 1 (no restrictions within the country but isolation from the rest of the world with travel restrictions in place). Level 1 continued into 2021. The largest region in the country, the greater Auckland area (home to about one-third of NZ’s population), experienced three more Level 3 lockdowns in August 2020 (lasting three weeks), February 2021 (three days), and late February/early March 2021 (one week) [2]; during these periods the rest of the country was at Level 2. The rapidity of the NZ initial response to the COVID-19 pandemic was internationally unprecedented and resulted in the elimination of community level COVID-19 and few COVID-19 related deaths [3,4].

The NZ gambling environment, although highly regulated, is relatively liberal, allowing many land-based opportunities for gambling, such as table games and electronic gaming machines (EGMs) in casinos, EGMs in community pubs and clubs, track and sports betting via the Totalisator Agency Board (TAB), lottery-based products (i.e., national lottery known as Lotto, instant scratch cards and keno), bingo, and more informal activities, such as charitable raffles [5]. Provision of online gambling, also known as remote interactive gambling, is prohibited in NZ, with the exception of government approved activities provided by the Lotteries Commission (i.e., lottery-based products) and the TAB. However, the NZ public is not prohibited from accessing overseas online gambling sites [6]. Although this means that a large selection of online gambling activities are accessible by the NZ public, online gambling by NZ adults occurs at relatively low rates. In 2015, 9% of adults gambled on online activities provided by the Lotteries Commission and the TAB, and 0.7% gambled on overseas sites [7]. By 2018, the proportion of adults who gambled on NZ-based online activities had increased slightly, to 13%, with 2% gambling on overseas sites [8]. Most of the online gambling was additional to gambling on the same activity via land-based means, with fewer than 10% gambling exclusively online, apart from keno gamblers where 18.5% solely gambled online [9]. This is similar to findings from other countries that have indicated that online gamblers generally also gamble on land-based activities [10].

During Alert Levels 3 and 4 in NZ, access to land-based gambling was denied with the only available gambling opportunities being online. These included NZ lottery-based products, gambling via the TAB and overseas online gambling sites. Preliminary gambling expenditure data collated by the NZ Department of Internal Affairs (the government department responsible for regulating gambling) indicated that during the 2019/20 financial year, all gambling expenditure decreased because of the pandemic, apart from lottery-based gambling, which increased by 13% due to increased online gambling [11]. Despite online gambling also being available through the TAB, because sports and racing events were cancelled, the opportunity for this type of betting was much reduced, leading to a 10% decrease in expenditure [11]. These data, however, only related to gambling expenditure on NZ regulated sites and did not capture overseas online gambling expenditure.

Several research articles investigating gambling behavior changes during COVID-19 restrictions worldwide have recently been published. These have mainly been cross-sectional online survey studies. A recent review of published studies relating to gambling participation during the COVID-19 pandemic identified that, as was to be expected with restricted land-based gambling opportunities, gambling engagement was reduced overall. However, risk factors for increased online gambling, although varying between studies, generally included a higher gambling risk categorization related to being a young adult, and being male [12]. Whilst informative, there were several methodological shortcomings with the studies discussed in that review, with major limitations being that the studies were conducted via online surveys (with inherent selection biases [13]) and relied on retrospective reports of gambling behaviors prior to COVID-19 lockdown restrictions.

The present study aimed to extend current knowledge of gambling behaviors during COVID-19 restrictions by examining NZ data. This was achieved via a new partial wave of data collection in 2020/21 from an existing population representative cohort for which annual gambling-related data had been collected over a four-year period, from 2012 to 2015; the NZ National Gambling Study. There were two aims to the present study: (1) to describe changes in NZ gamblers’ online gambling participation during a year in which there were COVID-19 lockdowns (Alert Levels 3 and 4), and (2) to use existing longitudinal data to identify risk factors associated with increased online gambling, because of COVID-19 lockdowns and the associated unavailability of land-based gambling opportunities.

## 2. Materials and Methods

### 2.1. Study Design

This study utilized data collected from the first and last years of the population representative NZ National Gambling Study (NGS). In 2012, NGS baseline data were collected from 6251 participants, including gamblers and non-gamblers. The design and methods of the original NGS are published separately [14]; the final data collection was in 2015 from 2770 of the original participants. An additional data collection was conducted in 2020/21 with selected NGS gambler participants. The 2020/21 data collection was designed for another study but, due to the pandemic, the opportunity was taken to include questions relating to gambling behaviors during COVID-19 lockdown periods.

### 2.2. Participants

NGS participants who scored as an at-risk gambler in 2012 or any of the subsequent data collection years (2013 to 2015) on either the Problem Gambling Severity Index (PGSI; score of 1+) [15] or the South Oaks Gambling Screen-Revised (SOGS-R; score of 3+) [16,17], who were contactable, had not withdrawn from the study, and for whom data were available at all data collection points, were invited to participate in 2020/21. This sample, whilst not population representative, is suitable for the current study, as early COVID-19 research has indicated that higher gambling risk categorization was a risk factor for increased online gambling during pandemic restrictions [12], and allows study of the behaviors of this at-risk population.

### 2.3. Procedure

The original NGS data were collected via face-to-face structured interviews; however, the 2020/21 data collection was via telephone structured interviews due to the various social isolation and restriction procedures in place at periods during the year. The 2020/21 questionnaire was modelled on the baseline NGS questionnaire to enable comparison and analyses of changes over time. However, the questionnaire was updated to take into consideration the changed gambling environment since 2012, including more detailed questions on online gambling and new questions relating to online gambling behavior change during periods of COVID-19 lockdown. The Short Gambling Harm screen was also added. Interviews were conducted from 2 October 2020 to 9 March 2021. Participants received a NZ $50 gift voucher after completion of the interview as an appreciation of the time they gave to the interview.

### 2.4. Measures

The 2020/21 questionnaire included the following measures.

*Gambling participation.* Participants were asked about their involvement in gambling activities in the past 12 months. For each reported activity, participants were asked about frequency and, if the activity was available online, these questions were repeated specifically for the online behavior. Participants were then asked if their online behavior increased, decreased or stayed the same during COVID-19 lockdown (Alert Levels 3 and 4). Participants were also asked about their involvement (participation and frequency) in free-to-play online gambling type activities (e.g., poker, casino games and bingo, not for money or prizes).

*Gambling risk level.* The nine-item Problem Gambling Severity Index [15] was used to measure current (past 12 months) gambling risk level. The PGSI has been assessed for robustness and reliability in the NZ population [18] and been found to have high internal reliability (Cronbach’s alpha of 0.86 or higher). Participants are categorized as non-gambler, non-problem gambler (score 0), low risk gambler (score 1 or 2), moderate risk gambler (score 3 to 7), or problem gambler (score 8 to 27).

*Gambling harm.* The 10-item Short Gambling Harm Screen (SGHS) [19] was used to measure experiences of gambling harm in the prior 12 months. The screen, adapted from a larger 72-item scale [20] measures financial, emotional/psychological and relationship harms. Each item scores 1 if the harm is present; the higher the score the more harm experienced by a participant.

*Major life events.* Participants were asked about 15 major life events (e.g., death of someone close, divorce, moving house) and whether they had experienced these in the prior five years (i.e., since the last data collection of the NGS in 2015). An ‘other’ category was also included.

*Mental health.* General psychological distress in the prior four weeks was measured with the 10-item Kessler-10 [21]. Scores ranged from 0 to 40 and were categorized as low distress (score 0 to 5), moderate distress (score 6 to 11), high distress (score 12 to 19), or severe distress (score 20 to 40).

*Quality of life.* The eight-item EUROHIS-QOL 8 measured quality of life in the prior two weeks. This short form is psychometrically robust, and overall performance is strongly correlated with scores from the original WHOQoL instrument [22]. Scores are presented as quartiles and categorized as very poor quality of life (score 0 to 21), poor (score 22 to 25), good (score 26 to 28) and very good (score 29 to 32).

*Substance use.* Hazardous alcohol consumption was measured using the three-item AUDIT-C, which was developed from the 10-item Alcohol Use Disorders Identification Test [23]. The AUDIT-C is time-efficient and accurate when compared with the full AUDIT [24]. Individual questions asked about tobacco and illicit drug use (most people who used illicit drugs reported using cannabis).

*General health.* Individual questions were asked about overall general health and disability in the prior 12 months.

*Deprivation.* To measure current (past 12 month) individual level of deprivation, the eight-item NZ Index of Socio-economic Deprivation for Individuals was used [25]. It has good statistical validity (Cronbach’s alpha 0.81). The more items that are scored positive, the greater the level of deprivation experienced by an individual.

*Sociodemographic data.* Sociodemographic data that could vary since the baseline survey were collected in the 2020/21 questionnaire (i.e., employment status, highest educational level, household composition, and annual personal income). Sociodemographic data collected at baseline and used in the present study included gender, age, ethnicity and country of birth.

### 2.5. Data Analysis

Descriptive statistics were produced for the variables of interest, presenting counts (*n*) and percentages (%). Inferential statistics, utilizing Pearson’s chi-square and Fisher’s exact tests, where relevant, were undertaken to examine changes over time.

Mixed effects logistic regression (repeated measures analysis) was undertaken to investigate associations with increased online gambling during lockdown. Potential explanatory covariates were considered for possible inclusion in the model. For age, gender and ethnicity, baseline (2012) measures were used. For other covariates that varied over time, both 2015 and 2020/21 measures were utilized.

Model selection generally proceeded through several steps. The first step identified candidate variables in bivariate analyses with the outcome variables that had a *p*-value ≤ 0.2. Models were then developed for each of the major data domains (e.g., demographics, gambling participation, co-existing conditions) using the candidate variables, in order to identify the best subset of variables from that data domain. All of the results from the separate domains were then considered for an overall model. Each of the model building procedures followed a stepwise selection method tempered by consideration of the information criteria. Parsimonious models were favored and competing models with a similar fit but markedly different compositions have all been reported.

The base odds and odds ratio of potential explanatory covariates are reported as point estimates and 95% confidence intervals, accompanied by a *p*-value for the covariate.

## 3. Results

### 3.1. Participant Demographic Characteristics

The study sample comprised 301 participants. There were slightly more females than males (56.5% female) and about two-thirds were born in NZ (69.8%). Half of the sample were of NZ European/other ethnicity (50.2%), one-quarter (24.6%) were of Māori ethnicity (NZ’s indigenous population), 14.3% were of Pacific ethnicity and 11% were of Asian ethnicity. At baseline (in 2012), two-thirds of participants were aged between 25 years and 54 years (64.1%). Table 1 details the participant demographic characteristics at baseline.

Table 2 details sociodemographic participant characteristics by data collection year. There were several characteristics that changed over time, with most of the differences noted in 2020/21 compared with 2012 and 2015. Overall, there was a tendency for higher annual personal income in 2020/21, probably due to most salaried participants receiving annual pay increases (*p* ˂ 0.0001). However, a majority of participants earned below or about the NZ national median income in 2020/21 and this remained similar to the percentage in 2012 (NZ median personal income was NZ$48,100 in 2020, and NZ$37,000 in 2012) [26].

There were increases in the percentages of people experiencing five or more major life events in 2020/21 (*p* ˂ 0.0001) and disability (*p* = 0.005). Whilst an increase in hazardous alcohol consumption was also noted in 2020/21 (*p* = 0.05), a lower proportion of participants reported using cannabis (*p* = 0.002).

Although statistical significance was attained for change in the highest educational level (*p* ˂ 0.0001), visual inspection indicated that the significance was likely due to the increase in participants who gained a university or ‘other’ qualification.

There were small, statistically non-significant changes in employment status, deprivation and quality of life. With small increases in the percentages of participants who were students/homemakers/retired or unemployed but actively seeking employment, there were corresponding decreases in percentages who were employed part-time or were unemployed and on a state benefit. Additionally, there were small decreases in the percentages of participants with a mid-level of deprivation and an increase in the percentage with good/very good quality of life.

There were no obvious major changes between 2020/21 and previous years for household composition, mental health, or general health.

### 3.2. Gambling Risk Level over Time

Participants moved between the different risk levels over time. At baseline in 2012, more than one-third (36.2%) had gambled in a risky manner (low risk, moderate risk or problem gambler); this increased to more than two-fifths (43.5%) in 2015. However, in 2020/21, there was a decrease in the proportion of risky gamblers with only one-quarter (25.6%) remaining classified as such (Table 3). This difference between the years was statistically significant using Pearson’s chi-squared test (*p* ˂ 0.0001).

### 3.3. Online Gambling Behavior during Lockdown

Table 4 details past-year online gambling participation and gambling behavior during periods of lockdown. The most popular activity (land-based/online), with 85% of the sample reporting participation was Lotto. Activities that were popular online, with half or more of the respondents who participated in the activity reporting online participation were keno (70%), Lotto (50%) and sports betting (50%), all provided by NZ operators. Online track betting and scratch card gambling were less popular, at 35% and 19%, respectively. For online Lotto and scratch card gambling, similar proportions either increased or decreased participation during lockdown with a larger proportion gambling at the same level as prior to lockdown (60% and 46% for Lotto and scratch cards, respectively). However, substantially higher proportions of participants reported decreased NZ-operated online sports and track betting during lockdown (50% and 45%, respectively) compared with the percentages who gambled more on these activities (8% and 14%, respectively). Similarly, a higher proportion of online keno participants decreased their gambling during lockdown (29%) compared with those who increased their participation (13%). Only 5% of participants reported engaging in overseas online gambling. Of these, one-third increased their gambling during lockdown and one-fifth reduced their gambling. Overall, for all online gambling activities (both NZ and non-NZ operated), about one-quarter of participants increased or decreased their online gambling during lockdown.

### 3.4. Risk Factors for Increased Online Gambling during Periods of COVID-19 Lockdown

Inferential analyses, adjusting for confounding factors, identified four covariates that were statistically associated with increased online gambling during COVID-19 lockdown periods (Alert Levels 3 and 4) in NZ. Table 5 details results of the bivariate and multiple logistic regression analyses.

There were two socio-demographic factors associated with increased online gambling. One was educational level, with participants with the highest educational level (university degree or higher) in 2020/21 having 8.07 times (adjusted) odds of increased online gambling, compared with participants without any formal qualifications. Almost one-quarter (22.8%) of participants with a university degree or higher increased their online gambling during lockdown. The other socio-demographic factor was hazardous alcohol consumption in 2015 but not 2020/21. Participants who drank alcohol in a hazardous manner in 2015 had 2.80 times (adjusted) odds of increased online gambling in lockdown, compared with participants who did not drink alcohol hazardously. Almost one-fifth of participants (18.8%) of participants who drank alcohol in a hazardous manner in 2015 increased their online gambling behavior during lockdown.

Being a risky gambler (low risk, moderate risk or problem gambler) in 2020/21 was associated with 4.09 times (adjusted) odds of increased online gambling compared with non-gamblers/non-problem gamblers. This association was not found with being a risky gambler in 2015.

Participants who engaged in free-to-play gambling-type activities in 2015 had 3.38 times (adjusted) odds of increased online gambling, compared with participants who did not participate in free-to-play gambling-type activities. This association was not found with engaging in free-to-play gambling-type activities in 2020/21. Almost one-quarter of participants (23.7%) of participants who engaged in free-to-play gambling-type activities in 2015 increased their online gambling behavior during lockdown.

No other covariates were associated with increased online gambling during lockdown, when confounding variables were taken into consideration, although some associations were noted in bivariate logistic regression analyses for age, employment status, cannabis use, experiencing two or more indicators of gambling harm and taking up gambling for money from a free-to-play site.

## 4. Discussion

The NZ population was relatively fortunate in that they were able to live much more of 2020/21 ‘normally’ compared with people in other countries who experienced longer lockdown restrictions due to COVID-19. Nonetheless, NZ experienced its fair share of strict lockdown periods during 2020/21 and this provided an opportunity to study gambling behavior changes during those times. A strength of the present study is that data from an existing population-representative gambling study (the NZ National Gambling Study) were available and could be used in analyses of COVID-19 related behavior change, with the opportunity taken to collect new data from the cohort in 2020/21. We were specifically interested in understanding changes in online gambling behavior during lockdown periods as land-based gambling was not possible at those times, and to understand current and past risk factors for increased online gambling during lockdown.

In 2020/21, online gambling participation was relatively high, ranging from 19% of scratch card gamblers to 70% of keno gamblers. In general, Lotto gamblers were the most common with half buying tickets online. Relatively few participants gambled on overseas online gambling sites with only 5% reporting doing so. Overall, 56% of participants reported gambling online. During periods of lockdown, two-thirds of the online gamblers reported that their online gambling stayed the same, and just over one-quarter reported a decrease in online gambling. This finding was similar to that reported in a scoping review conducted in the first part of 2021 [27], whereby it was reported that gambling behavior either stayed the same or decreased for most gamblers during the pandemic. The largest proportions of participants who reduced their online gambling during lockdown in the present study were those who gambled on track or sports events. This was unsurprising, given that most horse and dog race meetings and sports events in NZ and worldwide were cancelled during COVID-19 lockdown restrictions.

However, almost one-quarter of online gamblers increased their gambling during lockdown, with the largest proportions being for Lotto, scratch card gambling and overseas online gambling. Initially, this profile appeared to be different from other studies. For example, in Australia and Sweden, the largest increase in online gambling during lockdown was noted for track betting [28,29] and, as speculated by the Australian authors, was likely to be due to this gambling activity being the least affected by lockdown. In other words, the availability of online gambling drove the behavior. Thus, our results support that finding as in NZ, lottery products and overseas online gambling sites were the least affected by lockdown (i.e., they remained available), whilst NZ-based track events did not proceed. As keno is also provided by the Lotteries Commission, we expected a similar proportion of online keno gamblers to have increased their gambling activity during lockdown in comparison to those who decreased their online gambling. However, only 13% of online keno gamblers increased their gambling, whilst 29% reduced their gambling. This requires further research but one reason could be that keno appears to be substantially less popular in NZ as a lottery game compared with Lotto and scratch card gambling.

Other studies of online gambling behaviors during COVID-19 lockdowns have generally found that being male, a young adult and having a higher gambling risk level categorization were risk factors [12]. Our analyses also found that having a current risky gambling categorization (low risk/moderate risk/problem gambler) was significantly associated with increased online gambling during lockdown, although being a risky gambler in the past was not significantly associated. This supports prior research that has identified an association between current problem gambling behaviors and online gambling [30,31].

In our study, in bivariate analyses, although age was a risk factor for increased online gambling during lockdown, male gender was not, and the statistical significance for age disappeared when confounding factors were accounted for. This finding was contrary to that reported in many other studies. The reason for this difference bears further investigation as in NZ, online gamblers are more likely to be male and aged between 18 and 54 years [8]. However, gambling on NZ lottery products is the most popular online gambling activity [8]. NZ lottery gambling (land-based and online) is an activity participated in by relatively equal proportions of males and females [32] and this could be one explanation for male gender not being a risk factor for increased online gambling during lockdown in NZ, compared with other countries where online track betting was the most increased during lockdown. Track betting is traditionally participated in by males more than females.

However, our study found that highly educated participants (university degree or higher) had greater odds for increased online gambling in lockdown than uneducated participants. This could simply be due to financial stability. More highly educated people tend to earn higher incomes and to be able to work from home during periods of lockdown, compared with less educated people who may be in lower paid manual labor jobs and unable to work during lockdown, thus reducing disposable income for activities such as gambling. They may also more easily access individual (rather than shared) devices making it easier to gambling online. However, this is speculation and requires further investigation. As noted by [27], who reviewed 24 articles on gambling and COVID-19, knowledge on this subject is limited and unclear.

As previously mentioned, a strength of this study was the ability to draw upon previously collected data for the participants, thus reducing the likelihood of recall bias, which can occur when participants are asked to retrospectively reflect on behaviors. Our study found that participants who drank alcohol in a hazardous manner in 2015, or who engaged in free-to-play gambling type games in 2015 had higher odds for increasing their online gambling during lockdown in 2020/21. That current hazardous alcohol consumption was not associated with increased online gambling during lockdown is a similar finding to that noted by [33]. This is despite that fact that there was an increase in hazardous alcohol consumption amongst participants in the current study in 2020/21 compared with 2015. Nonetheless, it is interesting that in our study, prior hazardous alcohol consumption was associated with increased online gambling. There are several potential explanations including that during lockdown, alcohol was less easily available in NZ with only beer and wine available for purchase from supermarkets (the only retail stores that were open), although stronger liquor could be bought online and delivered. Furthermore, people were not able to socialize in alcohol-serving institutions such as pubs. Overall, a majority of the NZ population either reduced their alcohol consumption during lockdown or consumed alcohol as usual, with about one-fifth increasing alcohol consumption [34].

Whilst little studied, there is preliminary evidence that about one-quarter of free-to-play online gamers migrate to online gambling six months later, with micro-transactions being the only unique predictor of the migration [35]. In our study, such a migration could explain the risk factor of past participation in free-to-play gambling-type games being associated with increased online gambling in periods of lockdown, particularly if disposable income increased because of an inability to spend money on other activities. Bivariate analyses found that such a migration was associated with increased online gambling during lockdown; however, the statistical significance disappeared when confounding factors were accounted for, indicating that the association is complicated. This phenomenon requires further investigation, particularly since other researchers have not identified such a migration [36].

A limitation of our study is that, due to budget and time constraints, it was not possible to collect data from the full NGS cohort in 2020/21, with only participants who had previously scored as a risky gambler recruited. This means that our study is not population representative. However, collecting data only from people who had, at some point, previously been gambling in a risky manner is justifiable as this study aimed to investigate online gambling behaviors. Prior research has indicated that online gambling is associated with problem gambling [30,31] and earlier COVID-19 research indicated that higher gambling risk categorization was a risk factor for increased online gambling [12]. Thus, the present study enabled a detailed study of the behaviors of this at-risk population, which comprise a low percentage of a general population. Furthermore, only about one-quarter of participants were classified as a current risky gambler in 2020/21, which was a statistically significant reduction from earlier data collection years when there had been no disruptions to gambling accessibility and availability. A strength of the present study is that participants were not recruited via an online sample as is the case with the majority of previous studies investigating lockdown effects on online gambling. Online samples have their own limitations including careless responses and identity misrepresentation [37], and they are likely to under-represent people who do not regularly use online technology even though such people may partake in online gambling. However, online samples have benefits, such as the ability to obtain large samples of specific subgroups and the possibility of reduced bias for highly stigmatized topics [38].

## 5. Conclusions

To date, most studies of online gambling behaviors during periods of COVID-19 lockdown have been cross-sectional in nature. Whilst these have identified risk factors for increased gambling behaviors during periods of restriction and social isolation, colloquially known as lockdown, potential past behavior risk factors remained unknown. Yet knowing what such factors are could help in public health efforts to encourage safer online gambling behaviors during future periods of lockdown or other similarly stressful situations, especially as online gambling is known to be highly associated with risky and problematic gambling. This study identified that past hazardous alcohol consumption and past participation in free-to-play gambling-type games were both significantly associated with increased online gambling during lockdown. Being past behaviors, they are likely to be known to others and are potential trigger points for health providers and family or friends to check on a person’s gambling behaviors during lockdown and support safer gambling. The gambling activities in which the highest proportions of participants increased their gambling during lockdown were overseas online gambling and online scratch card gambling. Both types of activity are continuous in nature, and more likely to lead to harm than non-continuous activities such as Lotto gambling. Encouraging a person to stick to the recently released Lower Risk Gambling Guidelines [39] could help reduce potential harms from gambling during lockdowns or other extended periods when usual behaviors are not possible.

## Figures and Tables

**Table 1:** Participant demographic characteristics at baseline.

Characteristic	Category	*n* (%)
Gender	Male	131 (43.5)
	Female	170 (56.5)
Age (years) ^1^	18–24	21 (7.0)
	25–34	61 (20.3)
	35–44	63 (20.9)
	45–54	69 (22.9)
	55–64	48 (16.0)
	65+	39 (13.0)
Ethnicity	Māori	74 (24.6)
	Pacific	43 (14.3)
	Asian	33 (11.0)
	NZ European/Other ^2^	151 (50.2)
Country of birth	NZ	210 (69.8)
	Overseas	91 (30.2)

^1^ At the time of the 2020/21 interview these participants would have aged by eight years; ^2^ Other relates to any ethnicity that is not Māori, Pacific, Asian or NZ European.

**Table 2:** Participant sociodemographic characteristics by data collection year.

		2012	2015	2020/21	
Characteristic	Category	*n* (%)	*n* (%)	*n* (%)	*p*-Value
Employment status	Employed—Full time	136 (45.2)	144 (47.8)	139 (46.2)	0.07
	Employed—Part time	62 (20.6)	64 (21.3)	41 (13.6)	
	Unemployed—Looking ^1^	11 (3.7)	11 (3.7)	18 (6.0)	
	Unemployed—Benefit	28 (9.3)	23 (7.6)	20 (6.6)	
	Student/Home ^2^/Retired	62 (20.6)	58 (19.3)	83 (27.6)	
	Other	2 (0.7)	1 (0.3)	0 (-)	
Highest educational level	No formal qualification	131 (43.5)	117 (38.9)	116 (38.5)	˂0.0001 ^5^
	University degree	58 (19.3)	68 (22.6)	79 (26.2)	
	Vocational or trade	112 (37.2)	115 (38.2)	101 (33.6)	
	Other	0 (-)	1 (0.3)	5 (1.7)	
Household composition	Live alone	51 (16.9)	52 (17.3)	66 (21.9)	0.44
	Live with partner only	67 (22.3)	60 (19.9)	53 (17.6)	
	Live with partner + children	83 (27.6)	98 (32.6)	88 (29.2)	
	Other	100 (33.2)	91 (30.2)	94 (31.2)	
Annual personal income (NZ$)	Up to $20,000	94 (31.2)	84 (27.9)	66 (21.9)	˂0.0001
	$20,001 to $40,000	93 (30.9)	87 (28.9)	78 (25.9)	
	$40,001 to $60,000	56 (18.6)	69 (22.9)	52 (17.3)	
	$60,001 to $80,000	27 (9.0)	37 (12.3)	40 (13.3)	
	$80,001 to $100,000	14 (4.7)	12 (4.0)	22 (7.3)	
	$100,001+	7 (2.3)	7 (2.3)	8 (5.7)	
	Not reported	10 (3.3)	5 (1.7)	26 (8.6)	
Deprivation (score)	0	127 (42.2)	154 (51.2)	167 (55.5)	0.07
	1	72 (23.9)	58 (19.3)	68 (22.6)	
	2	41 (13.6)	31 (10.3)	28 (9.3)	
	3	19 (6.3)	20 (6.6)	15 (5.0)	
	4	20 (6.6)	19 (6.3)	9 (3.0)	
	5–8	78 (25.9)	78 (25.9)	101 (33.6)	
Quality of life (score; quartiles ^3^)	Very poor	92 (30.6)	95 (31.7)	83 (27.9)	0.09
	Poor	115 (38.2)	105 (35.0)	95 (31.9)	
	Good	66 (21.9)	63 (21.0)	69 (23.2)	
	Very good	28 (9.3)	37 (12.3)	51 (17.1)	
Major life events (number) ^4^	0	62 (20.6)	64 (21.3)	21 (7.0)	˂0.0001
	1	65 (21.6)	78 (25.9)	37 (12.3)	
	2	62 (20.6)	59 (19.6)	40 (13.3)	
	3	39 (13.0)	48 (15.9)	54 (17.9)	
	4	35 (11.6)	22 (7.3)	36 (12.0)	
	5+	38 (12.6)	30 (10.0)	113 (37.5)	
Mental health (psychological distress)	Low	169 (56.1)	184 (61.1)	163 (54.2)	0.53
	Moderate	72 (23.9)	72 (23.9)	85 (28.2)	
	High	43 (14.3)	33 (11.0)	37 (12.3)	
	Severe	17 (5.6)	12 (4.0)	16 (5.3)	
General health	Excellent	31 (10.3)	31 (10.3)	31 (10.3)	0.26
	Very Good	90 (29.9)	86 (28.6)	83 (27.6)	
	Good	113 (37.5)	113 (37.5)	104 (34.6)	
	Fair	58 (19.3)	53 (17.6)	56 (18.6)	
	Poor	9 (3.0)	18 (6.0)	27 (9.0)	
Disability	Yes	66 (21.9)	55 (18.3)	88 (29.2)	0.005
	No	235 (78.1)	246 (81.7)	213 (70.8)	
Hazardous alcohol use	Yes	115 (38.2)	96 (31.9)	124 (41.2)	0.05
	No	186 (61.8)	205 (68.1)	177 (58.8)	
Cannabis use	Yes	64 (21.3)	43 (14.3)	29 (9.6)	0.002
	No	237 (78.7)	258 (85.7)	272 (90.4)	

^1^ Unemployed—Looking refers to participants currently not working but actively seeking employment; ^2^ Home refers to participants in a full-time homemaker role; ^3^ Quartiles are based on the full baseline (2012) cohort; ^4^ Asked in a past year time frame in 2012 and 2015, and in a past 5-year timeframe in 2020/21; ^5^ Fisher’s exact test.

**Table 3:** Gambling risk level by data collection year.

	2012	2015	2020/21
Risk Level	*n* (%)	*n* (%)	*n* (%)
Non-gambler	13 (4.3)	7 (2.3)	0 (-)
Non-problem gambler	179 (59.5)	163 (54.2)	224 (74.4)
Low risk gambler	65 (21.6)	93 (30.9)	46 (15.3)
Moderate risk gambler	28 (9.3)	29 (9.6)	25 (8.3)
Problem gambler	16 (5.3)	9 (3.0)	6 (2.0)

**Table 4:** Participation in online gambling during lockdown.

Gambling Activity (Land-Based and Online)	Past Year Participation (*N* = 301)	Online Participation	Online Gambling during Lockdown		
			Increased	Same	Decreased
	*n* (%)	*n* (%)	*n* (%)	*n* (%)	*n* (%)
NZ Lotto	255 (85)	127 (50)	28 (22)	76 (60)	23 (18)
NZ Scratch card	134 (45)	26 (19)	7 (27)	12 (46)	7 (27)
NZ Keno	44 (15)	31 (70)	4 (13)	18 (58)	9 (29)
NZ Track	63 (21)	22 (35)	3 (14)	9 (41)	10 (45)
NZ Sports	24 (8)	12 (50)	1 (8)	5 (42)	6 (50)
Non-NZ online ^1^	15 (5)	15 (100)	5 (33) ^2^	8 (53) ^2^	3 (20) ^2^
All activities	271 (90)	153 (56)	37 (24)^2^	101 (66) ^2^	41 (27) ^2^

^1^ Overseas online activities included gambling for money on online poker and other card gambling; events and sports races; virtual casino table games, electronic gaming machines and bingo; buying a loot box/crate/lockbox in a video game; or any other online gambling activity; ^2^ Reported if true for any gambling activity, hence percentages total greater than 100%.

**Table 5:** Increased online gambling during lockdown—logistic regression results.

Covariate	Year	Category	*n*	Increased Online Gambling	Unadjusted (Bivariate)			Adjusted (Multi-Variate)		
					Odds Ratio	95% CI	*p*-Value	Odds Ratio	95% CI	*p*-Value
Gender	2012	Male	131	10.7%	0.77	(0.38, 1.55)				
		Female	170	13.5%	1.00	-	0.46			
Age (years) ^1^	2012	18–24	21	23.8%	1.00	-				
		25–34	61	21.3%	0.87	(0.27, 2.81)				
		35–44	63	14.3%	0.53	(0.16, 1.82)				
		45–54	69	11.6%	0.42	(0.12, 1.46)				
		55–64	87	2.3%	0.08	(0.01, 0.42)	0.02			
Ethnicity	2012	Māori	74	12.2%	0.91	(0.39, 2.1)				
		Pacific	43	4.7%	0.32	(0.07, 1.43)				
		Asian	33	18.2%	1.46	(0.53, 3.96)				
		NZ European/Other ^2^	151	13.2%	1.00	-	0.36			
Employment status	2015	Employed	208	13.9%	1.00	-				
		Unemployed	34	17.6%	1.32	(0.5, 3.47)				
		Other ^3^	59	3.4%	0.22	(0.05, 0.94)	0.09			
	2020/21	Employed	180	15.6%	1.00	-				
		Unemployed	38	15.8%	1.02	(0.39, 2.66)				
		Other	83	3.6%	0.20	(0.06, 0.69)	0.04			
Highest educational level	2015	No formal qualification	118	5.1%	1.00	-				
		Vocational or trade	115	12.2%	2.59	(0.96, 6.99)				
		University degree	68	25.0%	6.22	(2.32, 16.71)	0.001			
	2020/21	No formal qualification	121	5.8%	1.00	-				
		Vocational or trade	101	11.9%	2.20	(0.83, 5.81)		1.00	-	
		University degree	79	22.8%	4.81	(1.9, 12.14)	0.003	2.49	(0.87, 7.09)	0.003
Gambling risk level (PGSI)	2015	No risk ^4^	170	11.8%	1.00	-				
		At risk ^5^	131	13.0%	1.12	(0.56, 2.23)	0.75			
	2020/21	No risk ^4^	224	8.0%	1.00	-		1.00	-	
		At risk ^5^	77	24.7%	3.75	(1.85, 7.61)	0.0003	4.09	(1.90, 8.82)	0.0003
Gambling harm (SGHS)	2020/21	0	247	9.7%	1.00	-				
		1	28	17.9%	2.02	(0.70, 5.80)				
		2+	26	30.8%	4.13	(1.62, 10.50)	0.009			
Free-to-play gambling-type activities	2015	No	242	9.5%	1.00	-		1.00	-	
		Yes	59	23.7%	2.96	(1.42, 6.20)	0.004	3.38	(1.47, 7.76)	0.004
	2020/21	No	203	8.9%	1.00	-				
		Yes	98	19.4%	2.47	(1.23, 4.96)	0.01			
Gambled from FTP ^6^	2020/21	No	288	10.8%	1.00	-				
		Yes	12	50.0%	8.29	(2.52, 27.29)	0.0005			
Sought help for gambling	2015	No	295	12.5%	-	-				
		Yes	6	0.0%	-	-				
	2020/21	No	294	11.9%	1.00	-				
		Yes	7	28.6%	2.96	(0.55, 15.84)	0.20			
Hazardous alcohol use	2015	No	205	9.3%	1.00	-		1.00	-	
		Yes	96	18.8%	2.26	(1.13, 4.54)	0.02	2.80	(1.25, 6.24)	0.01
	2020/21	No	177	9.6%	1.00	-				
		Yes	124	16.1%	1.81	(0.91, 3.62)	0.09			
Cannabis use	2015	No	260	10.8%	1.00	-				
		Yes	41	22.0%	2.33	(1.01, 5.38)	0.05			
	2020/21	No	273	12.5%	1.00	-				
		Yes	28	10.7%	0.84	(0.24, 2.95)	0.79			

^1^ At the time of the 2020/21 interview these participants would have aged by eight years; ^2^ Other ethnicity relates to any ethnicity that is not Māori, Pacific, Asian or NZ European; ^3^ Other employment status refers to student/homemaker/retired/other; ^4^ No risk includes the PGSI categorizations of non-gambler and non-problem gambler; ^5^ At risk includes the PGSI categorizations of low risk, moderate risk and problem gambler; ^6^ FTP = Free-to-play.

## Data Availability

Microdata from the four years of the NGS are available on request at https://www.stats.govt.nz/integrated-data/apply-to-use-microdata-for-research/confidentialised-unit-record-files-curfs/ (accessed on 5 December 2021).
